# Don’t wait for the perfect moment: The national training program in family medicine in Angola

**DOI:** 10.4102/phcfm.v16i1.4458

**Published:** 2024-04-08

**Authors:** Israel C. Avelino, Kama Sandra M. Chimuco, Niurka T. Díaz, Adelson G. Jantsch

**Affiliations:** 1 College of General and Family Medicine, Order of Doctors of Angola, Angolan Medical Council, Luanda, Angola; 2 Hospital Municipal Olga Chaves, Lubango, Angola; 3 Huila Provincial Health Office, Lubango, Angola; 4 Universidade Aberta do SUS, Brasília, Brazil

**Keywords:** Angola, primary health care, family practice, healthcare workforce, Sub-Saharan Africa

## Abstract

Like many Sub-Saharan countries, Angola struggles with a shortage of trained health professionals, especially for primary care. In 2021, the Angolan Ministry of Health in collaboration with the Angolan Medical Council launched the National Program for the Expansion of Family Medicine as a long-term strategy for the provision, fixation and training of family physicians in community health centres. Of the 425 residents 411 (96.7%) who entered the programme in 2021 will get their diplomas in the following months and will be certified as family physicians. Three main aspects make this National Programme unique in the Angolan context: (1) the common effort and engagement of the Ministry of Health with the Angolan Medical Council and local health authorities in designing and implementing this programme; (2) decentralisation of the training sites, with residents in all 18 provinces, including in rural areas and (3) using community health centres as the main site of practice and training. Despite this undeniable success, many educational improvements must be made, such as expanding the use of new educational resources, methodologies and assessment tools, so that aspects related to knowledge, practical skills and professional attitudes can be better assessed. Moreover, the programme must invest in faculty development courses aiming to create the next generation of preceptors, so that all residents can have in every rotation one preceptor or tutor responsible for the supervision of their clinical activities, case discussions and sharing their clinical duties, both at community health centres and municipal hospitals.

## Introduction

In the current scenario of fast economic growth and social development, Angola still suffers from a shortage and poor distribution of healthcare professionals, especially in primary health care (PHC). With approximately 0.21 doctors per 1000 inhabitants, the country falls far behind the numbers of other Portuguese-speaking countries such as Brazil (2.4/1000) and Portugal (3.3/1000).^1^ Over the past 15 years, this situation has improved considerably, increasing from 0.06 in 2005 to the current 0.21, because of the expansion of medical training and the creation of new medical schools across the country.^1^ For many decades, the *Universidade Agostinho Neto* was the sole institution responsible for training doctors and graduated 60 physicians every year. Today, seven public and two private schools graduate more than 600 physicians every year.

There is a network of community health centres (CHC) and municipal hospitals responsible for providing PHC to the community, but several challenges hinder their effectiveness. These include inadequate infrastructure, governance issues, a shortage of essential medicines and supplies and a lack of trained healthcare personnel, particularly in rural and remote areas. As a result, Angolan PHC is only capable of managing a small set of health problems and a few programmatic health interventions.^2^ The current model of care is not organised in primary care teams responsible for a list of patients. Small teams of two or three professionals (mostly nurse technicians, some registered nurses and a few physicians) are responsible for running each of the programmatic health interventions, such as vaccination, child and maternal care, contraception, malnourishment screening and treatment and malaria treatment. Some CHCs can also treat tuberculosis, human immunodeficiency virus (HIV) and acquired immunodeficiency syndrome (AIDS). Most physicians are generalists with no training in family medicine (FM) and there are still some CHCs in small villages that have no physicians in their staff.

Achieving the sustainable development goals (SDGs) seems an impossible endeavour without investments from the Angolan state in promoting PHC as the main response to achieve Universal Health Coverage.^3,4^ Fixing these issues is not an easy task as it calls for thorough and enduring policies that must not only be written as official documents but implemented in the field.^5^

One solution to tackle this issue was to invest in FM as the medical specialty for PHC. At first, two public-private institutions – *Clínica Multiperfil* and *Clínica Endiama* – created a residency programme in FM in Luanda, in collaboration with the Ministry of Health of Angola (Angola-MoH). As a result, 40 new specialists were trained between 2015 and 2019. The success of this initiative led the Angola-MoH to launch a large-scale training programme for FM in 2019. After 3 years of successes and failures in putting in place a massive training program in a low-resource scenario, Angola has a lot to share with the academic community, policymakers and PHC providers regarding strategies to promote medical education in real-world scenarios. This short report aims to present the Angolan initiative to expand FM training nationally, its achievements to date, pitfalls on the run and the upcoming challenges.

## The national training programme in family medicine

The training programme was designed by the College of Family Physicians of the Angolan Medical Council. The existing curriculum running at the *Clínica Multiperfil* served as a pedagogical foundation for the National Programme. Adaptations were made for less advantaged provinces regarding mandatory rotations and the minimum required curriculum that a resident must experience during the whole course. Training sites were assessed by the Angola-MoH and the Angolan Medical Council, in collaboration with local health authorities, to ensure that the programme would be evenly implemented in all locations, including remote and rural areas. Ultimately, the decision to implement the programme in all municipalities was made by the Angola-MoH, with the main goal of providing medical coverage in every municipality in the country.

The 3 year curriculum, divided into a series of rotations in CHC and municipal hospitals, was designed with the aim of addressing the country’s epidemiological needs, training professionals to provide comprehensive, continuous and person-centred healthcare, while managing healthcare services and making rational use of resources. For this purpose, a set of essential skills was predefined for each rotation, and, in this way, the acquired competencies were continuously evaluated under the supervision and approval of the training supervisor in each rotation and at the end of each year of training. During rotations, residents have a logbook containing a list of tasks and procedures that need to be carried out and the number of times it needs to be performed during the rotation. After completing each task, residents must record them in their logbook, which is subsequently validated by their supervisor and the training advisor. The tasks are initially performed under the supervision of rotation supervisors and gradually residents gain independence to perform them autonomously.

The progression to the next year is contingent on performing all clinical activities, fulfilling all requirements in each rotation and obtaining a positive grade in the annual exam, which includes both practical and theoretical components. At the end of each year, residents are subjected to one practical exam, which includes a consultation with a real patient, followed by a case discussion, and one written exam covering content related to family medicine presented during the year. The resident’s final grade combines these two exams (60%) and the grades from all rotations (40%). If the resident has a final grade greater than 50%, he or she can be promoted to the next year of training. The title of specialist is awarded after the approval in the final exam in a public examination with an independent national jury, as mandatory by the National Institute of Specialisation in Health.

Cuban doctors from the Cuban cooperation with Angola were sent to each province to be responsible for the supervision of three to six residents in their daily clinical activities and to organise and provide weekly lectures and learning sessions. Throughout the 3 years of training, residents complete rotations lasting 4 to 6 weeks in various areas, including internal medicine, paediatrics, gynaecology and obstetrics, general surgery, orthopaedics, traumatology and dermatology. Most of their training happens at the CHC, where they provide mostly programmatic healthcare actions, such as prenatal care, childcare or contraception. This scenario is far from the ideal, and it limits the scope of their practice and narrows their learning experience, but it is the consequence of the current model of care established within the Angolan PHC.

While the aim of this article was not to conduct an in-depth analysis of Cuban cooperation and its influence on this training programme, we can assert that the presence of Cuban doctors is crucial today when there are hardly enough family physicians in Angola to train the new generation. Cuban family physicians are accustomed to working in challenging environments on missions in various countries, which broadens their perspective on the world and provides them with extensive clinical experience. The Cuban cooperation with Angola has a long history. Medical schools in Angola have been significantly influenced by the Cuban teaching model, with a strong emphasis on health promotion and disease prevention. On the other hand, this cooperation objectively focuses on training young family physicians. Training the future tutors in FM in Angola is a challenge that is still not high on the Angolan agenda.

### From national policy and strategic plan to real-world implementation

Several African countries grapple with significant hurdles in policy formulation and plan development because of limited capacity in these areas, poor stakeholder engagement and a dearth of up-to-date evidence to guide policymaking and planning. How to implement these policies and strategic plans in the real world adds another layer of complexity and creates a hurdle that often keeps policies confined to official documents.^5^ With its successes and failures, the National Program for the Expansion of FM in Angola managed to close the gap between policy design and real-world implementation, building the way for political commitment to become a change in practice.

In 2018, the World Health Organization presented its Operational Framework for PHC, describing 14 levers that must be pulled in order to improve PHC.^6^ One main aspect that must be shared with the readers is that this project pulled all four Core Strategic Levers at once to promote PHC (see Table 1). By examining each aspect of the National Programme in relation to its corresponding lever, one can understand why this policy has been successful in transforming the landscape, resulting in 10 times more family physicians being trained in all provinces in the last 3 years than what was trained only in Luanda over the last decade.

**TABLE 1:** Aspects of the National Programme for the Expansion of Family Medicine in Angola responsible by pulling the four core strategic levers from the WHO Operational Framework for primary health care.

Four core strategic severs	Description
Political commitment and leadership	PHC as the main strategy to promote UHC in the National Development Plan for Healthcare (2012–2025) and (2023–2027)^13,14^ Angola-MoH increasing the number of health workers to be recruited and facilitating the admission process in the country Angola-MoH leading the decision-making process for training medical specialties in the country.
Governance and policy frameworks	Angola-MoH and the Angolan Medical Council working in partnership designing and implementing the National Program for Family Medicine. Shared responsibilities regarding the implementation process: (a) the Angolan Medical Council responsible for designing the curriculum, delivering the educational programme and evaluating the residents; (b) the Angola-MoH responsible for financing the programme, hiring tutors from the Cuban cooperation, and evaluating the working process.
Funding and allocation of resources	Angola-MoH guaranteeing that residents and tutors will be paid in the CHC they will be allocated in large cities or in remote areas.
Engagement of communities and other stakeholders	Local authorities, district health managers and local representatives of the Angolan Medical Council engaged in the educational process of the residents. Local authorities playing a major role in facilitating the interaction between the communities and the CHC and supporting residents as part of the staff.

CHC, community health centres; Angola-MoH, Ministry of Health of Angola; PHC, primary health care.

Three main aspects make this National Programme unique in the Angolan context. The first aspect is the common effort and engagement of the Ministry of Health with the Angolan Medical Council and local health authorities in designing and implementing this programme. The second aspect is decentralisation. Training healthcare professionals in remote areas is a challenge even for richer countries such as Brazil, Canada and Australia.^7,8,9^ This is surely not a problem solved, but a first firm and consistent step has been taken in this direction. Finally, the third aspect is using CHC as the main site for training. Unlike many countries where FM training takes place in hospitals – Angola’s first two programmes in Luanda (*Clínica Multiperfil* and *Clínica Endiama*) are still hospital-based programmes – the National Programme places residents to work as ‘doctors in charge’ in CHC, close to where people live. Access to medical residency depends on approval in a public service entrance exam. Family medicine residents receive a slightly higher salary than polyclinic doctors without the specialised training.

## Challenges ahead

The National Programme will graduate its first cohort in the following months, and 411 of the 425 residents (96.7%) that entered the programme in 2021 will get their diploma and be certified as family physicians. Despite this undeniable success, many educational improvements must be made. Some of them are:

- The need for using new educational resources and methodologies beyond traditional books and lectures with slide shows.^10^
- To expand the range of assessment tools currently used, so that aspects related to knowledge, practical skills and professional attitudes can be better assessed.
- To invest in faculty development courses aiming to create the next generation of preceptors in FM.^11^
- To ensure that all residents have one preceptor or tutor responsible for the supervision of their clinical activities in every rotation. Including case discussions and sharing their clinical duties both at CHC and municipal hospitals.^12^

Table 2 summarises the strengths, weaknesses, opportunities, and threats currently perceived by the authors regarding the current scenario of the National Programme for the Expansion of FM.

**TABLE 2:** Strengths, Weakness, Opportunities and Threats Matrix (SWOT Matrix) describing the actual scenario after 3 years of implementation of the National Programme for the Expansion of Family Medicine, Angola, 2023.

**Strengths** Nationwide implementation of the programme in all 18 provinces. Decentralisation of family medicine training. Long-term strategic vision and programme sustainability. Community Health Centres as the primary training setting. Experienced and qualified preceptors. Short courses offered to residents covering a wide range of clinical topics. Robust training in dermatology to address the national gap in that specialty. Good infrastructure and well-equipped CHC and municipal hospitals. Routine accreditation process certifying CHC and municipal hospitals for teaching.
**Weaknesses** Excessive number of internships and courses in specialised care, outside the primary care setting. Excessive number of rotations and workload in specialised care, taking residents away from their CHC for weeks or even months. A lack of coordination between hospital -rotations and primary care practice, weakening patient and family care coordination. A lack of common teaching materials that all tutors can use in their activities – currently, the resources available are not adequate or are outdated, making each tutor responsible for preparing the materials for their own classes. This situation weakens the educational process and leads to heterogeneity within training sites. Educational resources often outdated or not contextualised to the Angolan scenario. A lack of a standardised theoretical and bibliographic framework for the entire programme that is suitable for the Angolan context. Limited educational resources, primarily consisting of slides and texts. Teaching methodologies limited to lectures, lacking strategies to develop specific skills and competencies. Despite having a list of objectives to be achieved in each internship, there is no structured competency-based curriculum in which learning objectives are supported by teaching methodologies focused on the development of each competency and assessed through specific methods to determine whether each competency has been achieved or not. A lack of faculty development activities to train residents to become the next generation of preceptors and teachers. A lack of continuous education for the actual tutors of the programme.
**Opportunities** National government’s political engagement in formulating, financing and implementing a comprehensive health workforce public policy. Angolan Medical Association is responsible not only for the governance of the training program, but also for designing it, verifying the educational suitability of institutions and overseeing the entire process. Long-standing Cuban cooperation, strengthening international ties. High local demand for family physicians in all areas, including patient care, management, teaching and research. New international collaborations within the Community of Portuguese Language Countries (CPLP) and South-South collaboration.
**Threats** Changes in national regulations could weaken the programme, its governance and financing. Its decentralisation may lead its educational outcomes too heterogeneous and hinder the assessment of all residents. Its decentralisation may challenge the accreditation process of the educational sites. Quality improvement measures may be difficult to implement if only the Angolan Medical Board were responsible for the decision-making process. Investments must be made to improve the current model of care, moving it away from selective PHC and expanding its scope of practice; otherwise the investments in medical education will not enough to improve the actual health needs of the community. Investments must be made to improve training for all health professionals working in primary care, that is nurses, technicians, dentists, managers, etc.

PHC, primary health care; CHC, community health centres.

## Conclusion

To effectively address our communities’ health needs, a more comprehensive approach is needed to improve PHC beyond FM. In other words, FM is just one element of the health workforce for PHC, and the health care workforce is only one lever out of the 14 that must be pulled to thoroughly promote PHC. Digital technologies, quality improvement strategies, expanding medicines and materials, payment strategies and research in PHC are some of the levers that are still waiting to be pulled in the Angolan PHC context. Moreover, the existing model of care must be modernised by expanding its scope of practice, making it less selective and only focused on programmatic health actions. These investments should not be limited to medical education but should include nurses, technicians, physiotherapists, dentists and health managers. No matter how competent family physicians are, they will not be able to change the Angolan health scenario without broad and comprehensive investments pulling all 14 levers.
